# Genome Sequence of *Leucobacter* sp*.* 4J7B1, a Plant-Osmoprotectant Soil Microorganism

**DOI:** 10.1128/genomeA.00398-15

**Published:** 2015-05-21

**Authors:** M. Manzanera, J. I. Vílchez, C. García-Fontana, C. Calvo, J. González-López

**Affiliations:** Institute for Water Research, and Department of Microbiology, University of Granada, Granada, Spain

## Abstract

We report the first genome sequence for *Leucobacter* sp. 4J7B1, a newly described desiccation-tolerant strain. The complete genome sequence of *Leucobacter* sp. 4J7B1 has been sequenced and is estimated to be around 3.5 Mb in size, with an average GC content of 62.18%. We predict 2,953 protein-coding sequences.

## GENOME ANNOUNCEMENT

The genus *Leucobacter* belongs to the class of high-GC-content, Gram-positive, nonsporulating Actinobacteria. Species of *Leucobacter* have been recovered from diverse ecological niches, including plants’ phyllosphere (1). *Leucobacter* sp. 4J7B1 is a desiccation-tolerant bacterium isolated from a *Nerium oleander* rhizosphere subjected to severe drought (2). Isolation of other desiccation-tolerant microorganisms from this environment, including the new species *Arthrobacter siccitolerans* 4J27, has been reported recently (3, 4). The production of xeroprotectants, such as glycerol, by many desiccation-tolerant microorganisms protects themselves against damage caused by drought and salts (5–11) and other stressors (12). Thus, the combination of soy plants and *Leucobacter* sp. 4J7B1 in the presence of 200-mM NaCl results in significant protection of the plant by the microorganism. This protection effect might be the result of glycerol production, a well-known osmoprotectant. To our knowledge, the complete genome sequence of the genus *Leucobacter* sp. 4J7B1 has not been deposited in the DDBJ/EMBL/GenBank databases. In this research, we determine the whole-genome sequence of *Leucobacter* sp. 4J7B1 with pyrosequencing technology as implemented at the 454 Life Science-Roche platform with a combined approach of shotgun and 8-Kb mate-pair sequencing (12).

A total of 150,575 reads were produced, with an average read length of 603 bases for the shotgun and 123,838 sequences with an average read length of 389,53 bases for the mate-pair sequencing strategy. The total number of sequenced bases is 129,339,181, representing a sequencing depth of around 38×. *De novo* assembly was performed with default parameters by using the Newbler version 2.6 assembler. The assembly resulted in 432 contigs, 149 of which were larger than 500 bp. The *N*_50_ of the contig assembly was 871,355 bp, and the largest contig was 1,030,920 bp. Most of these contigs were ordered in two scaffolds (based on mate-pair information), where the largest scaffold was 3,069,722 bp. This combination of scaffolds and contigs resulted in an estimated genome size of 3.5 Mb. Gap closure was attempted using gap-spanning clones and PCR products. Putative coding sequences were predicted and genes were annotated with a pipeline implemented at Lifesequencing S.L. (Valencia, Spain). Briefly, protein-coding sequences were predicted by the combined use of Glimmer (13–15), RNAmmer (16), tRNAScan (17, 18), and BLAST (19, 20). The complete genomic information for *Leucobacter* sp. 4J7B1 was found to contain 2,953 protein-coding genes, 5 rRNA operons, and 49 tRNA genes, with an average GC content for that chromosome of 62.18%.

Analysis of this genome sequence data led to propose the presence of several genes involved in glycerol metabolism in bacteria, such as *tagD*, *glpF*, or *glpQ1*, among others. This knowledge can lead to advance biotechnological applications in osmoprotection engineering (6, 8, 21).

In summary, the complete genome sequence of *Leucobacter* sp. 4J7B1 will contribute to improving the knowledge of plants’ osmoprotection by microorganisms.

### Nucleotide sequence accession numbers. 

The complete genome sequence of *Leucobacter* sp. 4J7B1 has been deposited in the TBL/EMBL/GenBank databases under accession numbers CDWJ01000001 to CDWJ01000432.
